# Prevalence of musculoskeletal disorders in patients with chronic dyspnea

**DOI:** 10.25122/jml-2025-0080

**Published:** 2025-05

**Authors:** Kinga Vindis, Katalin Babeș

**Affiliations:** 1 Doctoral School of Biomedical Sciences, Faculty of Medicine and Pharmacy, University of Oradea, Oradea, Romania; 2 Department of Psycho Neuroscience and Recovery, Faculty of Medicine and Pharmacy, University of Oradea, Oradea, Romania; 3 Cardiology Department, Faculty of Medicine and Pharmacy, University of Oradea, Oradea, Romania

**Keywords:** musculoskeletal disorders, chronic dyspnea, prevalence

## Abstract

Dyspnea, regardless of cause, is frequently associated with neuromuscular disorders. Many adults with chronic dyspnea associated with neuromuscular disorders have associated heart or lung disease. This study aimed to identify the association between chronic dyspnea of different etiologies and musculoskeletal disorders identified at presentation to establish a rehabilitation program. This prospective study was carried out in the Medical Rehabilitation Department of Dr. Pop Mircea Municipal Hospital in Marghita and included 163 consecutive patients with chronic dyspnea of different etiologies. The analysis showed no significant difference in the risk of musculoskeletal disorders between men and women (RR = 1.0090). Similarly, we found no significantly increased risk of musculoskeletal disorders in obese individuals compared to overweight (RR = 1.1223; 95% CI, 0.9648–1.3055; z = 1.495; *P* = 0.135) or normal-weight individuals (RR = 1.0399; 95% CI, 0.8997–1.2019; z = 0.529; *P* = 0.597). Overweight individuals also did not show a significantly increased risk compared to normal weight (RR = 1.0793; 95% CI, 0.9132–1.2756; z = 0.895; *P* = 0.371). The risk of developing head protrusion was 1.5 times higher in obese vs. normal-weight (RR = 1.4943; 95% CI, 1.0426–2.1416; z = 2.187; *P* = 0.029), and 1.4 times higher in overweight vs. normal-weight individuals (RR = 1.3565; 95% CI, 0.9153–2.0103; z = 1.519; *P* = 0.129). No significant difference in this risk was found between obese and overweight groups (RR = 1.1015; 95% CI, 0.8494–1.4286; z = 0.729; *P* = 0.466). As for thoracic kyphosis, we determined a 2.1-fold higher risk of occurrence in obese patients compared to normal-weight (RR = 2.1250; 95% CI, 1.2403–3.6408, z = 2.744; *P* = 0.006) and 2-fold higher in overweight compared to normal-weight (RR = 2.0357; 95% CI, 1.1812–3.5085; z = 2.560; *P* = 0.011). The study highlights the correlations between dyspnea, musculoskeletal status, and variations by gender and age, suggesting directions for personalized therapeutic interventions.

## INTRODUCTION

Dyspnea is a common symptom encountered in patients with neuromuscular disorders and has a wide range of etiologies, including cardiac, pulmonary, psychiatric, and musculoskeletal causes such as scoliosis [1]. Historically, between the 1960s and 1980s, the sensation of respiratory effort was believed to be the primary mechanism behind dyspnea [2]. However, more recent research has shown that dyspnea arises from a complex integration of signals from the respiratory muscles and central motor commands, similar to those involved in limb muscle exertion [3]. Examination is important for etiologic diagnosis. It is important to pay attention to muscle wasting, use of accessory respiratory muscles, neck movement difficulties, thoraco-abdominal mobility, dyspnea during conversation, and cardiac evaluation, including a detailed neurologic examination for differential diagnosis [4]. Many adults with chronic dyspnea associated with neuromuscular disorders present with associated cardiac or pulmonary disease. Symptoms such as dyspnea or restricted mobility are usually considered to be caused by these conditions without further investigation [5]. For this reason, a detailed history and a thorough physical examination are required. In acute exacerbations of chronic dyspnea, it is essential to consider possible additional pathophysiologic disorders. Diagnostic tools like spirometry and biomarker analysis may not consistently correlate with dyspnea severity and are not universally reliable. Consequently, treatment should focus on identifying and treating the underlying cause of the symptom. Once the cause has been addressed, it is important to manage any persistent physiologic disturbances such as hypoxemia or acidemia [3].

### The relationship between musculoskeletal dysfunction and chronic dyspnea

Skeletal muscle dysfunction is a significant problem in patients with chronic obstructive pulmonary disease (COPD), affecting both ventilatory and non-ventilatory muscles. It is an important comorbidity associated with poor quality of life and survival. Causes of muscle dysfunction include muscle atrophy, changes in muscle fiber type, metabolic alterations, and chest wall remodeling, which negatively impact patients' physical capacity, especially during COPD exacerbations [6]. Respiratory muscles constantly adapt to the changing demands of breathing by remodeling, trying to compensate for the imbalance between energy supply and demand to optimize pumping, minimizing energy expenditure according to pathology. As the disease progresses, respiratory muscle effort increases, and sarcomere length-tension relationships become disadvantageous, leading to muscle atrophy, reduced cross-sectional area, a shift towards anaerobic processes in bioenergetics, decreased circulatory perfusion and compromised nervous system activation. These chronic processes also contribute to the remodeling of other body parts, increasing morbidity and mortality [7]. A characteristic of COPD is pulmonary hyperinflation, which affects the respiratory muscles. Physical deconditioning from reduced activity also contributes to limb muscle weakness [8]. Patients with COPD frequently have musculoskeletal comorbidities. Recent research suggests that these patients may develop postural disorders, but the mechanisms underlying these changes are not well understood. Pulmonary hyperinflation, limitations of inspiratory muscle mechanics, and increased trunk muscle exertion due to respiratory workload are contributing factors. Also, decreased flexibility of the rib cage and soft tissue contractures caused by hypertonicity can lead to postural changes. Over 60% of patients experience cervicalgia back pain associated with chest wall pain, leading to decreased mobility at these levels. In addition, decreased peripheral muscle strength has been observed, which results in loss of functional performance and physical inactivity associated with the observed postural disturbances [9].

Thoracic hyperkyphosis, defined by an excessive curvature of the thoracic spine of more than 40°, is a condition that tends to worsen with age, with no uniform criteria for delineating hyperkyphosis from normal age-related changes. It is more common in women than in men. Normal angles of kyphosis range from 20° to 40° in youth, reaching values of 48°–50° in women and 44° in men in older populations [10]. The prevalence of hyperkyphosis increases with age in both women and men, being most prevalent in women aged 50–59 years. Estimates suggest that between 20% and 40% of people over 60 have the condition, and studies indicate an increase in the angle of kyphosis of about 9 degrees per decade, often beginning after age 40 [10]. Hyperkyphosis also develops more rapidly in women than in men, and risk factors include musculoskeletal, neuromuscular, and sensory disorders [11,12]. Excessive loads on the spine combined with insufficient muscle strength of the paravertebral muscles can accelerate the degenerative process of vertebral structures and may contribute to the onset of kyphosis. Daily activities performed in an improper posture, such as head protrusion and limited shoulder mobility, aggravate this malalignment. Anteroposterior radiographs are essential for examining the vertebral bodies, while lateral radiographs provide valuable information about the height of the vertebrae disc [13]. If necessary, further investigations (CT and MRI) can be performed. Forward head posture is associated with changes in the scapular girdle (scapulae in lateral rotation, internal rotation of the glenohumeral joint), increased upper thoracic kyphosis, decreased mid-cervical lordosis and accentuation of the curvature of the upper cervical spine. This involves shortening of the suboccipital muscles (cervical extensors) and lengthening of the prevertebral muscles (cervical flexors), which keeps the weight of the head forward of the line of gravity, thereby increasing the bending moment on the spine. Studies show a correlation between age and the severity of anterior head projection, with an average angle of 49 degrees in people aged 65 to 74, 41 degrees between 75 and 84, and 36 degrees in those aged 85 years and over. Head posture angle measurements indicate that a smaller angle reflects a more pronounced anterior projection of the head [14]. Muscle contractures are the shortening of muscles that prevent normal relaxation and can lead to joint deformity. They can cause intense pain, decreased muscle strength, and atrophy [15].

The study aimed to identify the association between chronic dyspnea of various etiologies and musculoskeletal disorders identified at initial patient presentation to inform the development of personalized rehabilitation programs.

## MATERIAL AND METHODS

### Study design

This prospective study was conducted in the Medical Recovery Department of the Dr. Pop Mircea Municipal Hospital in Marghita. A total of 163 consecutive patients presenting with chronic dyspnea of various etiologies were initially recruited.

### Inclusion/exclusion criteria

All adult patients diagnosed with chronic dyspnea from various causes were considered for inclusion. Stratification was based on each patient’s medical history, clinical examination, and spirometry testing performed using a Contec SP10 spirometer, following the American Thoracic Society/European Respiratory Society (ATS/ERS) guidelines. Patients were previously evaluated for baseline pathology and continued chronic medication without intervention, except in cases of decompensation. 163 patients with chronic dyspnea of different etiologies were initially recruited.

Exclusion criteria included age under 18 years, psychogenic dyspnea, decompensated comorbidities affecting rehabilitation, and dyspnea above level 5 on the modified Borg scale. After applying the exclusion criteria, 146 patients were included in the study.

### Study tools

All patients underwent clinical assessment for musculoskeletal disorders. Spinal alignment was evaluated through both clinical examination and radiographic imaging. Anterior projection angles of the head between 45° and 49° were used as criteria for identifying kyphosis, adjusted for patient age. Paravertebral muscle contractures were assessed through palpation and recorded as present or absent based on the examiner's subjective evaluation.

**Figure 1:**
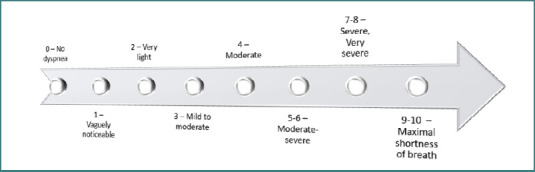
Modified Borg Scale

The degree of dyspnea was measured using the modified Borg dyspnea scale. This validated self-report tool quantifies the severity of breathlessness experienced during exercise. Although the results are subjective, the scale is considered reliable for assessing dyspnea among patients in clinical trials. It uses a scale from 0 to 10, where 0 indicates no dyspnoea and a score of 1–10 ranges from very mild symptoms to maximum shortness of breath (Figure 1) [16].

**Figure 2:**
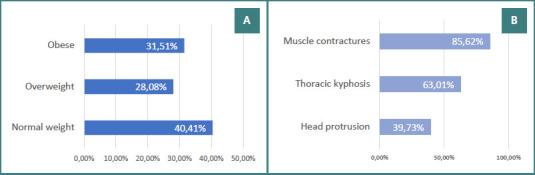
Distribution of patients by weight status and musculoskeletal findings. A, Distribution by weight category; B, Prevalence of anterior head projection, thoracic kyphosis, and muscle contracture.

### Sample size

We considered the following variables to determine the sample size: p - probability of occurrence, where 0 < p < 1; q - counter-probability, calculated as q = 1 - p; t - probability factor; x - error margin; N - total population size. We used the following formula to determine the sample size (*n*): *n* = t^2^ pq / (x^2^ + t^2^ pq / N.) Based on these calculations, we determined that the sample size (*n*) required for our study was 85.

### Statistical analysis

Data analysis was conducted using IBM SPSS Statistics for Windows, version 29.0 (IBM Corporation, Armonk, NY, USA). Categorical (nominal) variables were presented as frequencies and percentages, while continuous variables were expressed as means and standard deviations (SD). The Risk Ratio (RR) was used to assess the likelihood of developing musculoskeletal disorders in patients exposed to specific risk factors using the formula: RR = [Risk in exposed group] / [Risk in unexposed group] ([17]).

## RESULTS

### General cohort characteristics

The mean age of the patients in the study was 58.19 ± 7.58 years. Of the participants, 63.70% were men, and the majority (75.35%) were from urban areas. More than half of the patients (56.85%) were actively employed. Approximately 60% of the subjects had a high body mass index (BMI), and 8.90% of the patients were dependent on activities of daily living (ADL). Etiologically, 61.65% (*n* = 90) of patients had pulmonary disease, 29.45% (*n* = 43) had cardiac pathology, and the remaining 8.90% (*n* = 13) presented with other causes. According to the modified Borg dyspnea scale, 17.80% (*n* = 26) scored level 1, 44.52% (*n* = 65) scored level 2, and 37.67% (*n* = 55) scored level 3 [1]. The distribution according to BMI and the presence of musculoskeletal disorders are illustrated in Figure 2AB.

### Prevalence of musculoskeletal disorders in patients with chronic dyspnea by sex

Data analysis in Table 1 suggests a higher prevalence of musculoskeletal disorders in male patients (54.80% vs. 31.50%).

**Table 1:** Prevalence of musculoskeletal disorders in patients with chronic dyspnea according to sex

Parameter	Sex	With condition	Without condition
Musculoskeletal disorders	Female, *n* (%)	46 (31.50)	7 (4.80)
	Male, *n* (%)	80 (54.80)	13 (8.90)
Head protrusion	Female, *n* (%)	17 (11.64)	7 (4.80)
	Male, *n* (%)	41 (28.08)	13 (8.90)
Thoracic kyphosis	Female, *n* (%)	31 (21.23)	7 (4.80)
	Male, *n* (%)	61 (41.78)	13 (8.90)
Muscle contractures	Female, *n* (%)	46 (31.50)	7 (4.80)
	Male, *n* (%)	79 (54.10)	13 (8.90)

Risk analysis indicated no statistically significant difference in the likelihood of developing musculoskeletal conditions between women and men within the study cohort. The RR = 1.0090 value indicates that the risk of musculoskeletal disorders was similar between women and men. The 95% confidence interval (0.8831–1.1527) supports the absence of statistically significant differences between the two groups. The statistic (z = 0.131) indicates a very small value, suggesting that the observed difference was not significant. The *P* = 0.896 was much higher than 0.05, which means there was not enough statistical evidence to reject the null hypothesis (which, in this case, states that there was no difference between the risks of musculoskeletal disorders in women and men). No significant sex-based differences were found in the prevalence of head protrusion (RR = 1.0719; 95% CI, 0.7961–1.4432; z = 0.458; *P* = 0.647), thoracic kyphosis (RR = 1.0105; 95% CI, 0.8406–1.2147; z = 0.111; *P* = 0.912), or muscle contractures (RR = 1.0107; 95% CI, 0.8842–1.1554; z = 0.157; *P* = 0.876).

### Age-related prevalence of musculoskeletal disorders

Data analysis in Table 2 indicates a clear trend of increasing prevalence of musculoskeletal conditions with advancing age. The highest proportion was observed in individuals aged 51–60 (36.99%), while the lowest was recorded in those under 50 (15.06%).

**Table 2:** Prevalence of musculoskeletal disorders by age

Parameter	Age	With condition	Without condition
Musculoskeletal disorders	<50 years, *n* (%)	22 (15.06)	4 (2.73)
	51–60 years, *n* (%)	54 (36.99)	5 (3.42)
	>60 years, *n* (%)	50 (34.24)	11 (7.53)
Head protrusion	<50 years, *n* (%)	11 (7.53)	4 (2.73)
	51–60 years, *n* (%)	23 (15.75)	5 (3.42)
	>60 years, *n* (%)	24 (16.43)	11 (7.53)
Thoracic kyphosis	<50 years, *n* (%)	17 (11.64)	4 (2.73)
	51–60 years, *n* (%)	41 (28.08)	5 (3.42)
	>60 years,*n* (%)	34 (23.28)	11 (7.53)
Muscle contractures	<50 years, *n* (%)	22 (15.06)	4 (2.73)
	51–60 years, *n* (%)	54 (36.99)	5 (3.42)
	>60 years, *n* (%)	49 (33.56)	11 (7.53)

Risk analysis did not reveal a significantly higher likelihood of musculoskeletal changes in individuals aged 51–60 years compared to those under 50 years (RR = 1.0962; 95% CI, 0.9187–1.3080; z = 1.019; *P* = 0.308) or those over 60 years (RR = 1.1316; 95% CI, 0.9888–1.2951; z = 1.796; *P* = 0.073). Similarly, no significant difference was observed between individuals under 50 and over 60 (RR = 1.0323; 95% CI, 0.8437–1.2631; z = 0.309; *P* = 0.757).

In terms of head protrusion, no significant differences in risk were found between patients aged 51–60 years and those under 50 years (RR = 1.1201; 95% CI, 0.7888–1.5906; z = 0.634; *P* = 0.526) or between those under 50 and over 60 years (RR = 1.0694; 95% CI, 0.7323–1.5618; z = 0.347; *P* = 0.728). In contrast, the risk was 1.2 times higher in patients aged 51–60 years compared to those aged >60 years (RR = 1.1979; 95% CI, 0.9026–1.5899; z = 1.250; *P* = 0.211). Regarding thoracic kyphosis, the risk of occurrence was comparable in patients aged 51–60 years with those aged <50 years (RR = 1.1010; 95% CI, 0.8742–1.3867; z = 0.818; *P* = 0.414) and in those aged <50 years compared with those aged >60 years (RR = 1.0714; 95% CI, 0.8213–1.3977; z = 0.509; *P* = 0.611). A 1.2-fold increase in risk was observed in the 51–60 age group compared to those over 60 (RR = 1.1797; 95% CI, 0.9712–1.4328; z = 1.666; *P* = 0.096), but this also did not reach statistical significance. We did not detect higher risks of developing muscle contractures in patients aged 51–60 years compared to those less than 50 years (RR = 1.0817; 95% CI, 0.9022–1.2968; z = 0.848; *P* = 0.396) or in those aged >60 years (RR = 1.1207; 95% CI, 0.9715-1.2928; z = 1.564; *P* = 0.118).

### Prevalence of musculoskeletal disorders according to weight status

Analysis of the data in Table 3 suggests that people with normal weight had a higher prevalence of musculoskeletal disorders (32.88%) than overweight (24.66%) and obese (28.76%) patients, respectively. The prevalence of head protrusion was highest in obese patients (17.12%), followed by overweight (12.32%) and normal weight (10.27%). A similar prevalence was also found for thoracic kyphosis (23.28% vs. 20.54% vs. 5.4%). Similar to general musculoskeletal disorders, muscle contractures were more common in normal-weight individuals.

**Table 3:** Prevalence of musculoskeletal disorders according to weight status

Parameter	Weight status	With condition	Without condition
Musculoskeletal disorders	Normal weight, *n* (%)	48 (32.88)	11 (7.53)
	Overweight, *n* (%)	36 (24.66)	5 (3.42)
	Obese, *n* (%)	42 (28.76)	4 (2.73)
Head protrusion	Normal weight, *n* (%)	15 (10.27)	11 (7.53)
	Overweight, *n* (%)	18 (12.32)	5 (3.42)
	Obese, *n* (%)	25 (17.12)	4 (2.73)
Thoracic kyphosis	Normal weight, *n* (%)	8 (5.47)	11 (7.53)
	Overweight, *n* (%)	30 (20.54)	5 (3.42)
	Obese, *n* (%)	34 (23.28)	4 (2.73)
Muscle contractures	Normal weight, *n* (%)	47 (32.19)	11 (7.53)
	Overweight, *n* (%)	36 (24.66)	5 (3.42)
	Obese, *n* (%)	42 28.76	4 (2.73)

We did not determine a higher risk of musculoskeletal disorders in normal weight than in overweight or obese (RR = 1.1223; 95%CI, 0.9648–1.3055; z = 1.495; *P* = 0.135, respectively RR = 1.0399; 95%CI, 0.8997–1.2019; z = 0.529; *P* = 0.597), but neither in overweight compared to normal weight (RR = 1.0793; 95%CI, 0.9132–1.2756; z = 0.895; *P* = 0.371). The risk of developing head protrusion was 1.5 times higher in obese vs. normal-weight (RR = 1.4943; 95%CI, 1.0426–2.1416; z = 2.187; *P* = 0.029) and 1.4 times higher in overweight vs. normal-weight (RR = 1.3565; 95%CI, 0.9153–2.0103; z = 1.519; *P* = 0.129), but the risk was not higher in obese than overweight (RR = 1.1015; 95%CI, 0.8494–1.4286; z = 0.729; *P* = 0.466). As for thoracic kyphosis, we determined a 2.1-fold higher risk of occurrence in obese patients compared to normal-weight (RR = 2.1250; 95%CI, 1.2403–3.6408; z = 2.744; *P* = 0.006) and 2-fold higher in overweight compared to normal-weight (RR = 2.0357; 95%CI, 1.1812–3.5085; z = 2.560; *P* = 0.011), but no higher risk in obese versus overweight patients (RR = 1.0439; 95%CI, 0.8774–1.2419; z = 0.484; *P* = 0.628). In terms of muscle contractures, we did not detect higher risks of occurrence in obese versus normal- or overweight patients (RR = 1.1267; 95%CI, 0.9667–1.3132; z = 1.527; *P* = 0.127, respectively RR = 1.0399; 95%CI, 0.8997–1.2019; z = 0.529; *P* = 0.597) and neither in overweight versus normal weight (RR = 1.0835; 95%CI, 0.9152–1.2829; z = 0.931; *P* = 0.352).

### Prevalence of musculoskeletal disorders according to the cause of chronic dyspnea

Analysis of the data in Table 4 reveals that patients with pulmonary pathology had a significantly higher prevalence of musculoskeletal disorders (31.36%) compared to those with cardiac-related dyspnea (13.70%). Across all assessed categories (head protrusion, thoracic kyphosis, and muscle contractures), musculoskeletal abnormalities were more frequently observed in patients with chronic dyspnea of pulmonary origin.

**Table 4:** Prevalence of musculoskeletal disorders by cause of dyspnea

Parameter	Etiology	With condition	Without condition
Musculoskeletal disorders	Pulmonary, *n* (%)	75 (31.36)	15 (10.27)
	Cardiac, *n* (%)	20 (13.70)	3 (2.05)
	Other causes, *n* (%)	31 (21.23)	2 (1.36)
Head protrusion	Pulmonary, *n* (%)	29 (19.86)	15 (10.27)
	Cardiac, *n* (%)	9 (6.16)	3 (2.05)
	Other causes, *n* (%)	20 (13.70)	2 (1.36)
Thoracic kyphosis	Pulmonary, *n* (%)	49 (33.56)	15 (10.27)
	Cardiac, *n* (%)	14 (9.58)	3 (2.05)
	Other causes, *n* (%)	29 (19.86)	2 (1.36)
Muscle contractures	Pulmonary, *n* (%)	74 (50.68)	15 (10.27)
	Cardiac, *n* (%)	20 (13.70)	3 (2.05)
	Other causes, *n* (%)	31 (21.23)	2 (1.36)

No statistically significant difference was found in the risk of musculoskeletal disorders in patients with chronic dyspnea due to other causes compared to those with dyspnea of pulmonary (RR = 1.1273; 95%CI, 0.9931–1.2795; z = 1.854; *P* = 0.064) or cardiac etiology (RR = 1.0803; 95%CI, 0.9019–1.2939; z = 0.839; *P* = 0.402). Similarly, no significant difference was observed between patients with cardiac-related dyspnea and those with pulmonary-related dyspnea (RR = 1.0435; 95%CI, 0.8687–1.2534; z = 0.455; *P* = 0.649). A significantly higher risk of head protrusion was identified in patients with dyspnea of other etiologies than those with pulmonary causes (RR = 1.3793; 95%CI, 1.0739–1.7715; z = 2.519; *P* = 0.012). The risk was also elevated compared to cardiac causes (RR = 1.2121; 95%CI, 0.8521–1.7242; z = 1.070; *P* = 0.285), though this difference was not statistically significant. No significant difference in head protrusion risk was found between the cardiac and pulmonary groups (RR = 1.1379; 95%CI, 0.7707–1.6802; z = 0.650; *P* = 0.516)

Regarding thoracic kyphosis, patients with chronic dyspnea due to other causes had a significantly higher risk of occurrence compared to those with pulmonary-related dyspnea (RR = 1.2219; 95%CI, 1.0370–1.4397; z = 2.394; *P* = 0.017). No significant differences in risk were identified between patients with dyspnea of other causes and those with cardiac-related dyspnea (RR = 1.1359; 95%CI, 0.8947–1.4422; z = 1.047; *P* = 0.295), nor between patients with cardiac and pulmonary causes of dyspnea (RR = 1.0756; 95%CI, 0.8306–1.3929; z = 0.553; *P* = 0.580).

In terms of muscle contractures, no significantly increased risk was observed in patients with chronic dyspnea of other causes compared to those with pulmonary (RR = 1.1298; 95%CI, 0.9946–1.2835; z = 1.876; *P* = 0.061) or cardiac etiology (RR = 1.0803; 95%CI, 0.9019–1.2939; z = 0.839; *P* = 0.402). Similarly, there was no significant difference in the risk of muscle contractures between patients with cardiac and pulmonary causes of dyspnea (RR = 1.0458; 95%CI, 0.8702–1.2569; z = 0.478; *P* = 0.633).

### Prevalence of musculoskeletal disorders in patients with chronic dyspnea according to dyspnea severity

As shown in Table 5, musculoskeletal disorders were present in over 80% of patients, with the highest prevalence observed in those with a Borg score of 2 (39.72%) and 3 (30.82%), and the lowest in those with a Borg score of 1 (15.75%). A similar trend was noted for head protrusion, thoracic kyphosis, and muscle contractures, with the highest prevalence consistently found in patients with Borg scores of 2 and 3.

**Table 5:** Prevalence of musculoskeletal disorders according to dyspnea severity (modified Borg scale)

Parameter	Borg Score	With condition	Without condition
Musculoskeletal disorders	Borg score 1, *n* (%)	23 (15.75)	3 (2.05)
	Borg score 2, *n* (%)	58 (39.72)	7 (4.80)
	Borg score 3, *n* (%)	45 (30.82)	10 (6.85)
Head protrusion	Borg score 1, *n* (%)	9 (6.16)	3 (2.05)
	Borg score 2, *n* (%)	25 (17.12)	7 (4.80)
	Borg score 3, *n* (%)	24 (16.44)	10 (6.85)
Thoracic kyphosis	Borg score 1, *n* (%)	21 (14.38)	3 (2.05)
	Borg score 2, *n* (%)	40 (27.39)	7 (4.80)
	Borg score 3, *n* (%)	31 (21.23)	10 (6.85)
Muscle contractures	Borg score 1, *n* (%)	23 (15.75)	3 (2.05)
	Borg score 2, *n* (%)	58 (39.72)	7 (4.80)
	Borg score 3, *n* (%)	44 (30.13)	10 (6.85)

Despite these observed distributions, statistical analysis revealed no significant differences in the risk of musculoskeletal disorders across Borg categories. The risk of musculoskeletal disorders in patients with Borg score 2 was not significantly higher compared to those with Borg score 1 (RR = 1.0087; 95%CI, 0.8574–1.1867; *z* = 0.104; *p* = 0.917) or Borg score 3 (RR = 1.0906; 95%CI, 0.9382–1.2677; *z* = 1.129; *P* = 0.259). Similarly, no difference was found between Borg score 1 and score 3 (RR = 1.0812; 95%CI, 0.8972–1.3029; *z* = 0.820; *P* = 0.412).

We determined no higher risk of developing head protrusion in patients with Borg score 2 compared to those with Borg score 1 or Borg score 3 (RR = 1.0417; 95%CI, 0.7162–1.5150; z = 0.214, *P* = 0.831; RR = 1.1068; 95%CI, 0.8331–1.4704; z = 0.700, *P* = 0.484) and neither in patients with Borg score 1 compared to those with Borg score 3 (RR = 1.0625; 95%CI, 0.7178–1.5727; z = 0.303, *P* = 0.762).

We did not determine a higher risk of developing thoracic kyphosis in patients with Borg score 1 compared to those with Borg score 2 or Borg score 3 (RR = 1.0281; 95%CI, 0.8478–1.2467; z = 0.282; *P* = 0.778, respectively (RR = 1.1473; 95%CI, 0.9191–1.4571; z = 1.242; *P* = 0.214) and neither in patients with Borg score 2 compared to those with Borg score 3 (RR = 1.1256; 95%CI, 0.9115–1.3900; z = 1.099; *P* = 0.272).

We did not determine a higher risk of developing thoracic kyphosis in patients with Borg score 2 compared to those with Borg score 1 or Borg score 3 (RR = 1.0087; 95%CI, 0.8574–1.1867; z = 0.104; *P* = 0.917, respectively (RR = 1.0951; 95%CI, 0.9401–1.2757; z = 1.167; *P* = 0.243) and in patients with Borg score 1 compared to those with Borg score 3 (RR = 1.0857; 95%CI, 0.8994–1.3106; z = 0.856; *P* = 0.392).

## DISCUSSION

The study aimed to identify the association between chronic dyspnea of different etiologies and musculoskeletal disorders identified at the time of patient presentation for the establishment of a rehabilitation program. The most common etiologic factors were pulmonary, followed by cardiac factors and obesity. Also, the age of patients was associated with an increased prevalence of chronic dyspnea [18]. The systematic review by Muller and coworkers [18] suggests a higher prevalence among women. Studies by Gut-Gobert *et al*. [19] emphasize significant physiological differences in breathing between men and women, contributing to a higher predisposition of women to dyspnea [20]. These differences, known as dysanapsis [21], manifest as narrower airway diameters and thicker bronchial walls, which require more intense work of breathing to ensure adequate airflow. In addition, the influence of sex hormones, such as progesterone and estrogen, on respiratory mechanisms suggests that biological factors play a crucial role in how women perceive and experience dyspnea. Recent studies conducted by Brown *et al*. [22] add another dimension to this issue by exploring the link between trait self-monitoring and self-reported dyspnea sensation, which may provide valuable insights into understanding and managing this condition in relation to gender [18]. In our study, male patients predominated (over 60%), which can be explained by the fact that patients were mainly referred for a non-specific rehabilitation program rather than for dyspnea reduction.

Analysis of the data from our study suggests that men had a higher prevalence of musculoskeletal disorders (kyphosis and anterior head projection) compared to women, in contrast to other studies suggesting a higher prevalence of hyperkyphosis in women [10]. Kazman *et al*. [23] suggested that thoracic kyphosis was more pronounced in women than men, similar to Gonçalves *et al*. [24]. This may suggest a difference in health risks between the two sexes, possibly influenced by biological, behavioral, or environmental factors.

A clear trend has been noted that as age increases, the prevalence of musculoskeletal disorders, head protrusion, thoracic kyphosis, and muscle contractures increases significantly. This indicates a correlation between advancing age and the development of these conditions, suggesting the need for increased attention to musculoskeletal health in older people, similar to the data in the literature [18]. Analysis of risk for musculoskeletal changes by age group showed no significant increase in risk for patients aged 51–60 years compared to those under 50 or over 60 years. Also, no significant differences in anterior head projection and thoracic kyphosis were observed between the age groups analyzed. One explanation could be the determined mean age and daily activities of the patients included in the study. The study published by Müller *et al*. supports an accentuation of kyphosis in the 50–59 age range [18].

In terms of muscle contractures, the risk was not significantly higher in patients aged 51–60 years compared to those in other age groups. Dyspnea is associated with weight gain [25]. It was determined that the prevalence of musculoskeletal disorders was significantly higher among people with normal weight compared to overweight or obese individuals. The prevalence of thoracic kyphosis was also found to increase significantly with increasing body mass index. The results also suggest an increasing trend of head protrusion with increasing weight. It is a common postural problem, aggravated by contemporary lifestyle, and its severity tends to increase with advancing age [13]. Musculoskeletal disorders, head protrusion, thoracic kyphosis, and muscle contractures are more common in patients with chronic dyspnea of pulmonary cause, an aspect supported by other studies.

The level of dyspnea, assessed on the Borg scale, was higher in patients with chronic cardiopulmonary dyspnea. The prevalence of musculoskeletal disorders was higher in patients with chronic cardiopulmonary dyspnea. An inverse correlation was observed between the angle of thoracic kyphosis and diaphragmatic mobility. In addition, women diagnosed with COPD have a significantly higher thoracic kyphosis angle compared to men with the same condition [26].

## CONCLUSION

Pulmonary pathology emerged as the most common cause of dyspnea, followed by cardiac conditions and obesity. Age and gender significantly influenced the prevalence of dyspnea, with women being more frequently affected, possibly due to physiological and hormonal factors. There was also a higher prevalence of musculoskeletal conditions, such as kyphosis and anterior head protrusion, among men. As age increases, these conditions become more common, indicating the need to monitor musculoskeletal health in older people. Normal-weight patients also had a higher prevalence of musculoskeletal disorders compared with overweight or obese patients. The study highlights the correlations between dyspnea, musculoskeletal status, and variations by gender and age, suggesting directions for personalized therapeutic interventions.

## Data Availability

The original contributions presented in this study are included in the article. Further inquiries can be directed to the corresponding author.

## Personal thanks

The authors thank the University of Oradea for considering the logistic facilities they have used.
